# Neuroendoscopic lavage versus external ventricular drainage in spontaneous intraventricular hemorrhage: A cohort-matched comparative study

**DOI:** 10.1016/j.bas.2026.106149

**Published:** 2026-06-23

**Authors:** Tomáš Radovnický, Karel Pištěk, Martin Sameš

**Affiliations:** a Department of Neurosurgery, Masaryk Hospital, Faculty of Health Studies, Jan Evangelista Purkyne University in Usti nad Labem, Usti nad Labem, Czech Republic; b Second Faculty of Medicine, Charles University, Czech Republic

**Keywords:** Intraventricular hemorrhage, Neuroendoscopy, External ventricular drainage, Hydrocephalus

## Abstract

**Introduction:**

Spontaneous intraventricular hemorrhage (IVH) has high mortality and poor functional outcome. External ventricular drainage (EVD) is standard treatment but is limited by catheter obstruction, infection, prolonged intensive care, and shunt-dependent hydrocephalus. We compared neuroendoscopic lavage (ENDO) with EVD alone in adults with spontaneous IVH.

**Research question:**

Does neuroendoscopic lavage offer advantages over external ventricular drainage alone in IVH management?

**Methods:**

This matched study included 46 adults with spontaneous IVH (23 ENDO + EVD; 23 EVD). Patients were matched 1:1 for age, admission Glasgow Coma Scale (GCS), and modified Graeb score (mGS). Outcomes were ventricular clot reduction, ICU stay, EVD duration, shunt dependency within 6 months, and modified Rankin Scale (mRS) at discharge and 90 days.

**Results:**

Baseline characteristics were comparable. Endoscopic lavage achieved a 66.7 % reduction in ventricular blood burden. Median EVD duration was significantly shorter after ENDO (3 vs. 7 days) as was ICU stay (8 vs. 25 days). Shunt placement was necessary in 13 % (ENDO) versus 26 % (EVD). Among shunted patients, time to VP shunt was shorter after ENDO (10.0 vs. 15.5 days). Functional outcome favored ENDO at discharge (median mRS 4 vs. 5) and at 90 days (median mRS 3 vs. 5). Ninety-day favorable outcome (mRS 0-3) was achieved in 52.1 % versus 13.1 %, respectively.

**Conclusion:**

Neuroendoscopic lavage in spontaneous IVH is associated with improved clot clearance, reduced ICU and drainage duration, lower shunt dependency, and superior early functional outcome compared with EVD alone. Prospective randomized trials are needed to confirm these findings.

## Highlights

• Endoscopic lavage shortens external ventricular drainage (EVD) duration compared with only EVD treatment.
• Intensive care unit stay is significantly shorter after endoscopic lavage treatment.
• Endoscopic lavage shows a trend toward lower ventriculoperitoneal shunt dependency.
• Early clot removal may improve cerebrospinal fluid pathway restoration in IVH.
• Functional outcome at discharge favored the endoscopic treatment group.

## Graphical abstract

**Figure:**
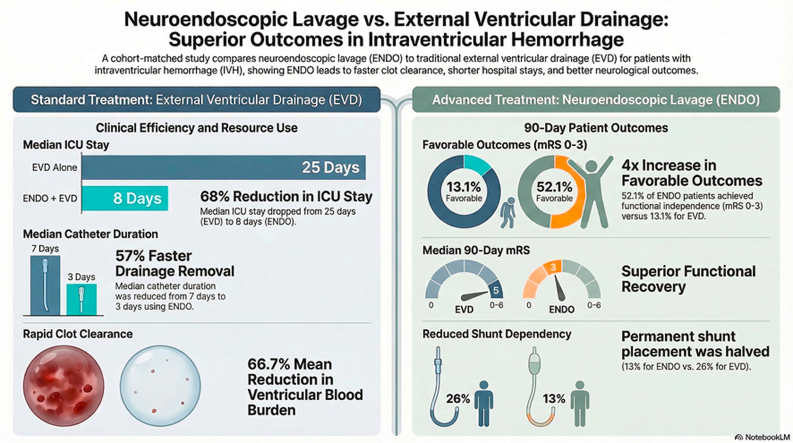

## 1 Introduction

Spontaneous intraventricular hemorrhage (IVH) complicates 10 - 40 % of intracerebral hemorrhages and carries mortality rates approaching 50 - 80 % (Qureshi et al., 2009; Huttner et al., 2006). Cerebrospinal-fluid (CSF) diversion with external ventricular drainage (EVD) remains the gold standard of acute management, yet catheter occlusion by clot, risk of infection, prolonged intensive-care stay, and a high incidence of chronic hydrocephalus limits its effectiveness (Mahto et al., 2023). Pharmacological clot lysis with intraventricular alteplase reduced ventricular blood burden but failed to show improvement of functional outcome (Hanley et al., 2017).

Neuro-endoscopic evacuation has emerged as an alternative, offering direct clot removal and the opportunity to restore CSF pathways. Initial case series demonstrated substantial reductions in ventricular blood volume and shorter catheter dwell times (Basaldella et al., 2012; Longatti et al., 2004), but conflicting data exist regarding shunt dependence and neurological recovery (Fiorindi et al., 2022). Moreover, many comparative studies are limited by unmatched controls or heterogeneous inclusion criteria, obscuring the true clinical benefit of an endoscopic strategy.

Against this background, we conducted a single-center, cohort-matched study to compare neuro-endoscopic lavage with EVD in adults with spontaneous IVH and small-volume (<30 ml) intracerebral hemorrhage. We hypothesized that meticulous endoscopic clot evacuation would: (i) enhance radiological clearance of intraventricular blood, (ii) shorten intensive-care stay and EVD duration, (iii) reduce risk of permanent hydrocephalus, and (iv) translate into superior early functional outcome. By applying strict 1:1 matching on key prognostic variables (age, admission Glasgow Coma Scale and modified Graeb score (Morgan et al., 2013)), we aimed to minimize confounding and provide a clearer assessment of the therapeutic value of neuro-endoscopic lavage in this challenging patient population.

## 2 Materials and methods

### 2.1 Study design and setting

We performed a single-center, retrospective, cohort-matched study. Consecutive adult patients treated for spontaneous intraventricular hemorrhage (IVH) between 2018 and 2024 were screened from the prospectively maintained institutional database.

### 2.2 Patient selection

All identified patients were evaluated against predefined eligibility criteria.

#### 2.2.1 Inclusion criteria

• Age ≥18 years
• Spontaneous primary IVH or secondary IVH extending from supratentorial intracerebral hemorrhage (ICH) with parenchymal volume ≤30 ml
• Definitive treatment with either
  o external ventricular drainage alone (EVD cohort), or
  o neuroendoscopic lavage followed by EVD (ENDO cohort)
• Availability of complete clinical, radiological, and 90-day follow-up data

#### 2.2.2 Exclusion criteria

• Pediatric patients (<18 years)
• Missing critical imaging or follow-up information
• Concomitant major intracranial pathology that could confound primary outcomes (e.g. territorial cerebral infarction, traumatic brain injury, aneurysmal SAH)

A total of 46 patients met the eligibility criteria - 23 treated with EVD alone and 23 who underwent endoscopic intraventricular lavage followed by EVD. The 23 endoscopically treated patients represented all consecutive ENDO procedures performed during the study period. The EVD control cohort was selected from a larger institutional EVD registry using 1:1 matching. Although some EVD-treated patients preceded the introduction of routine endoscopic lavage, the EVD cohort was not purely historical, as several patients were treated during the same period as the ENDO cohort. Throughout the study period, both groups were managed within the same department according to the same institutional protocols for EVD management, neurocritical care, imaging follow-up, and assessment of permanent CSF diversion. Matching was performed with nearest-neighbour methodology (without replacement) on age, admission Glasgow Coma Scale score, and pre-operative modified Graeb score. Because all patients underwent surgical intervention within 24 h after admission, admission GCS was considered representative of early pre-operative neurological status and was used for matching.

## 3 Surgical techniques

### 3.1 External ventricular drainage

External ventricular drains were inserted according to our long-standing institutional standard. A frontal burr-hole was made at Kocher's point on the side of the more dilated lateral ventricle identified on pre-operative imaging. After insertion, the drainage system was levelled 10 cm H_2_O above the external auditory meatus and left fully open. Catheter position was verified with a CT scan on postoperative day 1. Subsequent surveillance CTs guided a stepwise weaning protocol once the intraventricular clot volume had decreased. Patients who remained drainage dependent after CSF clearance underwent definitive diversion with a ventriculo-peritoneal shunt.

### 3.2 Endoscopic ventricular lavage

Neuro-endoscopic lavage was performed through a frontal burr-hole positioned 3 cm lateral to the midline at the coronal suture on the side of the more dilated lateral ventricle. After trepanation, intra-operative ultrasound confirmed the ventricular trajectory and depth for endoscope insertion. A 30° rigid neuro-endoscope (Minop®, Aesculap AG, Tuttlingen, Germany) was introduced, and continuous irrigation with sterile, body-temperature (37 °C) isotonic saline was commenced. Intraventricular hematoma clots were sequentially aspirated under direct vision during ongoing irrigation. When all accessible clot within the ipsilateral lateral ventricle had been removed, the endoscope was advanced into the third ventricle to evacuate additional hematoma. After anatomical landmarks were clearly visualized, an endoscopic third ventriculostomy (ETV) was performed to prevent obstructive hydrocephalus from possible residual dorsal third-ventricle and fourth ventricle clot. A septostomy was subsequently performed to access and aspirate any clots in the contralateral lateral ventricle. At the end of the lavage, an external ventricular drain (EVD) was inserted through the same burr-hole and initially clamped. Intracranial pressure was recorded at 6, 12, 24, and 48 h post-procedure in the intensive-care unit. If pressures remained stable and surveillance CT scans showed no ventricular enlargement (Evans’ index ≤0.30), the EVD was removed. Patients who demonstrated persistent ventricular dilatation with elevated pressure and suitable cerebrospinal-fluid parameters underwent ventriculo-peritoneal shunt placement.

### 3.3 Data collection

Demographic data (age, sex), comorbidities, admission neurological status (GCS), radiological variables and treatment timings were extracted from electronic medical records.

• Radiology. Admission and immediate post-procedural CT scans were reviewed by two independent raters (blinded to treatment) to determine
  o Change in mGS (ΔmGS pre-operative – post-operative)
  o ICH volume via the ABC/2 method
  o Hydrocephalus Evans' index.
  o Inter-rater disagreements >1 point in mGS were resolved by consensus.
• Clinical outcomes.
  o Modified Rankin Scale (mRS) at hospital discharge and 90 days.
  o Duration of intensive-care stay (days).
  o Duration of EVD in situ (days).
  o Need for permanent CSF diversion (ventriculo-peritoneal shunt within 6 months).

### 3.4 Statistical analysis

Normality of continuous variables was assessed using the Shapiro–Wilk test. Normally distributed variables are presented as mean ± standard deviation and were compared using Student's *t*-test. Non-normally distributed or ordinal variables are presented as median (interquartile range) and were compared using the Mann–Whitney *U* test. Categorical variables are reported as counts and percentages. Effect sizes with 95 % confidence intervals were reported for all key outcomes. For non-normally distributed continuous or ordinal variables, between-group differences were quantified using the Hodges–Lehmann estimator with corresponding confidence intervals. Functional outcome measured by the modified Rankin Scale (mRS) was analysed using proportional-odds logistic regression and expressed as common odds ratios (cOR). Time-to-event data for ventriculo-peritoneal shunt implantation were analysed using Kaplan–Meier estimates and compared with the log-rank test; hazard ratios (HR) with 95 % confidence intervals were reported. All statistical tests were two-tailed, and *p* < 0.05 was considered statistically significant. After matching, the two cohorts were analysed as independent groups by admission Glasgow Coma Scale score and modified Graeb score.

## 4 Results

### 4.1 Patient cohort

Between 2018 and 2024, 46 consecutive patients fulfilled all eligibility criteria and were matched on age, admission Glasgow Coma Scale (GCS) and pre-operative modified Graeb (mGraeb) score. Twenty-three patients underwent external ventricular drainage alone (EVD group) and 23 underwent neuro-endoscopic lavage followed by EVD (ENDO group). Complete radiological and clinical follow-up was available for every patient.

### 4.2 Baseline characteristics

Baseline demographics, comorbidities (Charlson comorbidity index - CCI) and hemorrhage severity were comparable between groups (Table 1).

**Table 1:** Baseline characteristics.

Variable	EVD (n = 23)	ENDO (n = 23)	*p*-value
Age, years – mean (SD)	64.3 (13.6)	63.6 (17.4)	0.86
Admission GCS – mean (SD)	7.3 (3.1)	6.7 (2.5)	0.46
CCI – median (IQR)	2 (0-5)	2 (0-7)	0.74
mGraeb score – mean (SD)	16.5 (4.8)	18.4 (5.8)	0.23

Regarding hemorrhage etiology, all included patients had spontaneous IVH, either primary IVH or IVH associated with a small periventricular supratentorial ICH ≤30 ml. No aneurysmal, arteriovenous malformation-related, traumatic, tumor-related, or other structural cause of IVH was identified in either group. All patients in both groups underwent the initial surgical intervention within 24 h after hospital admission.

### 4.3 Peri-operative course

Neuro-endoscopic lavage achieved a mean postoperative decrease of 13 points (SD 5.8) in the modified Graeb score, it means 66.7 %.

Median time with the ventricular catheter in situ was 7 days (IQR 4.5-12.5) in the EVD group versus 3 days (IQR 2-4) after endoscopic lavage (HL = 4, 95 % CI (Huttner et al., 2006; Fiorindi et al., 2022); *p* < 0.001).

Endoscopic lavage was associated with a markedly shorter need for intensive care: median 8 days (IQR 6.5-14) compared with 25 days (IQR 5-30) for EVD alone (HL = 12, 95 % CI [0, 22]; *p* = 0.002). (Table 2).

**Table 2:** ICU stay and catether in situ time (days).

Variable	EVD	ENDO	Effect size (HL)	*p-*value
ICU stay - median (IQR)	25 (5-30)	8 (6.5-14)	12, 95 % CI [0, 22]	0.002
Catether in situ - median (IQR)	7 (4.5-12.5)	3 (2-4)	4, 95 % CI [2, 7]	0.001

Procedure-related complications were infrequent. Meningitis occurred in 1 patient in the ENDO group and in 3 patients in the EVD group. No catheter misplacement, procedure-related rebleeding, or other clinically relevant treatment-related complication was recorded in either group.

### 4.4 Shunt implantation

Persistent hydrocephalus requiring ventriculo-peritoneal shunt placement developed in 3 of 23 patients (13 %) in the ENDO group, compared with 6 of 23 patients (26 %) in the EVD cohort. The risk of VP shunt implantation was lower after endoscopic lavage (HR 0.51, 95 % CI 0.13-2.04; p = 0.340), however, this difference did not reach statistical significance. The small number of shunt events (n = 9) limits the precision of this estimate. Among patients who required shunting, the median time to VP shunt was 10.0 (IQR 8.0-11.0) days in the ENDO group versus 15.5 (IQR 14.3-18.3) days in the EVD group (HL = 6.5, 95 % CI [1, 16]; p = 0.03).

### 4.5 Functional outcomes

Functional outcome consistently favored the ENDO cohort at both time points (Table 3, Table 4, Table 5). At hospital discharge, median mRS was significantly lower in the endoscopic group (4 [IQR 3-5]) compared with the EVD group (5 [IQR 5-6]; HL = 1, 95 % CI (Qureshi et al., 2009; Huttner et al., 2006), cOR = 9.63, 95 % CI [1.07, 86.18] p < 0.001). A shift toward better functional outcomes was observed, with 30.4 % of ENDO patients achieving mRS 0–3 at discharge versus 4.3 % in the EVD cohort, and mortality (mRS 6) was lower in the ENDO group (8.7 % vs. 34.8 %).

**Table 3:** Functional outcome (modified Rankin scale).

Outcome	EVD – median (IQR)	ENDO – median (IQR)	Effect size (HL)	Common odds ratio	*p*-value
mRS at discharge	5 (5-6)	4 (3-5)	1, 95 % CI [1, 2]	9.63, 95 % CI [1.07, 86.18]	<0.001
mRS at 90 days	5 (4-6)	3 (2.5-4.5)	2, 95 % CI [0, 2]	6.67, 95 % CI [1.56, 28,52]	0.005

**Table 4:** Distribution of mRS at discharge.

mRS at discharge	ENDO group	EVD group
0 - 1	8.7 %	0 %
2 - 3	21.7 %	4.3 %
4 - 5	60.9 %	60.9 %
6	8.7 %	34.8 %

**Table 5:** Distribution of mRS at 90 days.

mRS at 90 days	ENDO group	EVD group
0-1	13 %	4.4 %
2-3	39.1 %	8.7 %
4-5	26.1 %	39.1 %
6	21.7 %	47.8 %

At 90 days, this difference became more pronounced. Median mRS improved to 3 (IQR 2.5-4.5) in the ENDO group compared with 5 (IQR 4-6) in the EVD group (HL = 2, 95 % CI [0, 2]; cOR = 6.67, 95 % CI [1.56, 28,52], p = 0.005). Favorable functional outcome (mRS 0–3) was achieved in 52.1 % of ENDO patients versus 13.1 % of EVD-treated patients. Mortality at 90 days remained lower in the ENDO cohort (21.7 % vs. 47.8 %).

Overall, these data demonstrate a clinically meaningful and statistically significant shift toward improved early functional recovery following neuroendoscopic lavage.

## 5 Discussion

Neuro-endoscopic lavage was associated with markedly better functional outcome than EVD alone. Median modified Rankin Scale at discharge improved from 5 in the EVD cohort to 4 after endoscopic treatment, a difference that reached strong statistical significance (*p* < 0.001). The benefit widened over time: at 90 days the ENDO group achieved a median mRS of 3 compared with 5 in the EVD group (*p* = 0.005). A two-point shift on the mRS represents a clinically meaningful improvement - from severe dependency to moderate disability - underscoring the potential of endoscopic clot evacuation to accelerate neurological recovery after intraventricular hemorrhage. However, based on our data, it is still clear that IVH is a very serious diagnosis, and the prognosis is poor, whether IVH is treated endoscopically or with EVD. This early functional benefit is larger than, and partly contrasts with, the results of most prior comparative studies. Basaldella et al. analysed 96 patients with massive IVH and reported that flexible-scope aspiration did not influence 1-year mRS when compared with historical EVD controls (Basaldella et al., 2012). Longatti et al. observed that 38 % of endoscopically treated patients were fully independent (mRS 0–1) at 12 months. This study lacked an EVD comparison group (Longatti et al., 2004). When benchmarked against our 90-day median mRS 3, these data suggest that the early gains we report can translate into durable independence for a substantial subset of patients. Sarti et al. compared an “endoscopic brain-wash” technique with EVD in 45 patients and found a non-significant trend toward better long-term outcome in the endoscopy arm (mRS ≤3 in 41.6 % vs 33.3 %) (Sarti et al., 2024).

Persistent hydrocephalus required ventriculo-peritoneal shunting in 13 % of endoscopically treated patients compared with 26 % after EVD alone. Although absolute numbers are small, the relative 50 % risk reduction aligns closely with the 2-fold higher shunt risk for EVD reported by Basaldella et al. (17 % vs 50 %) (Longatti et al., 2004) and the pooled relative risk of 1.93 (95 % CI 1.28–2.92) derived from five comparative studies in the Fiorindi meta-analysis (Fiorindi et al., 2022). Importantly, our mean time-to-shunt was 9 days after lavage versus 16 days in controls, suggesting benefit of early clot evacuation to provide adequate CSF clearance.

The endoscopy cohort in our study has a ventricular catheter in situ for a median period of 3 days versus 7 days after EVD. This corelates with shorter ICU stay in the ENDO group. Endoscopic patients left intensive care significantly earlier - 8 days compared with 25 days for EVD alone. This exceeds the 6- to 7-day advantage reported by Chen et al. (11 ± 5 vs 18 ± 7 days) and the 6-day mean reduction aggregated in the Fiorindi meta-analysis (12 vs 18.5 days) (Fiorindi et al., 2022; Chen et al., 2011).

Despite these promising results, questions remain regarding the optimum timing, patient selection, and technique. Advocates propose early surgical intervention, generally within 24 - 72 h of hemorrhage for patients with large-volume intraventricular clots and deteriorating neurological status (Qureshi et al., 2009; Sarti et al., 2024). On the other hand, overly aggressive or untimely interventions in unstable patients may pose heightened risks of rebleeding or intraoperative complications. Furthermore, variations in endoscopic device selection (rigid vs flexible) and use of adjunctive methods (e.g., fibrinolytic infusions post-evacuation) complicate the interpretation of outcomes. As illustrated by multiple single-center series, the overall success also depends on institutional expertise, patient co-morbidities, and hemorrhage etiology (Mahto et al., 2023; Basaldella et al., 2012).

The 66.7 % reduction of intraventricular blood clot we achieved is on par with the best flexible-scope experience reported by Longatti (64 %) (Longatti et al., 2004), despite the inherent reach limitation of rigid optics. The endoscopic approach is commonly referred to as “lavage,” but this term does not fully reflect the scope of the procedure. Beyond clot irrigation and removal, endoscopy also enables at least partial restoration of physiological cerebrospinal fluid (CSF) pathways. This additional effect may represent a key mechanism underlying the potential superiority of endoscopic treatment over external ventricular drainage (EVD), which provides CSF diversion but does not directly re-establish normal intraventricular CSF circulation. Our EVD protocol used a drainage level of 10 cm H_2_O above the external auditory meatus. It could be argued that a lower drainage setting, such as 0 cm H_2_O, might have promoted more rapid passive drainage of bloody CSF and thereby improved outcomes in the EVD-only group. However, our institutional approach favours controlled CSF diversion rather than maximal drainage, aiming to avoid overdrainage and to maintain at least partial physiological CSF circulation and resorption. This strategy was also intended to reduce prolonged drainage dependency and the risk of permanent hydrocephalus, although this assumption cannot be proven from the present data. Therefore, differences in EVD height settings should be considered when comparing EVD-based strategies across studies. Complication rates were low in both cohorts. Although meningitis was numerically less frequent after ENDO + EVD than after EVD alone, the number of events was too small to allow meaningful statistical interpretation.

### 5.1 Study limitations

**Several caveats temper these conclusions**

1. Retrospective, single-centre design. Although strict 1:1 matching minimised baseline imbalance, unrecognised confounders cannot be excluded.
2. Sample size. With 46 patients, the study is under-powered for rare complications or for multivariable modelling of long-term outcomes.
3. Although treatment allocation was mainly determined by the availability of an endoscopically trained surgeon rather than by formal selection criteria, this remains a potential source of bias. Surgeon availability and the progressive introduction of the endoscopic technique may have influenced which patients underwent ENDO + EVD. Matching for age, admission GCS, and preoperative modified Graeb score reduced imbalance in major prognostic variables, but residual selection bias cannot be excluded.
4. Follow-up horizon. Functional status was assessed at 90 days; longer follow-up may detect late convergence between groups.
5. Technique heterogeneity in literature. Differences in endoscope type (rigid vs flexible), use of fibrinolytics, and adjunctive procedures (ETV, septostomy) limit direct comparison across studies.

## 6 Conclusion

This matched cohort study suggests that neuroendoscopic lavage is associated with improved ventricular clot clearance, shorter ICU stay, reduced EVD duration, and better early functional outcome compared with EVD alone in spontaneous IVH. Although the reduction in shunt dependency did not reach statistical significance, the direction of effect favored endoscopic treatment. These findings support the concept that early clot removal and restoration of CSF pathways may positively influence early recovery. Prospective multicenter randomized trials are required to confirm these observations and define optimal patient selection.

## Disclosure

The authors declare that they have no conflict of interest.

All authors have approved the final article.

## Contribution of authors

Radovnický T. – surgical treatment, database management, preparing the manuscript. Pištěk K. – surgical treatment. Sameš M. – coordination of the project.

## Funding

This study was supported by Internal Grant Agency of Krajska zdravotni, a.s., grant identification IGA-KZ-2025-1-12.

## Declaration of competing interest

The authors declare that they have no known competing financial interests or personal relationships that could have appeared to influence the work reported in this paper.
